# Pre-Professional International Mobility of European Pharmacy Students—A French Example

**DOI:** 10.3390/pharmacy11020059

**Published:** 2023-03-20

**Authors:** Mihayl Varbanov, Danièle Bensoussan, Marc Devocelle

**Affiliations:** 1 Université de Lorraine, CNRS, L2CM, F-54000, Nancy, France; 2 Laboratoire de Virologie, CHRU de Nancy Brabois, F-54500 Vandoeuvre-Les-Nancy, France; 3 UTCT (Unité de Thérapie Cellulaire et banque de Tissus), Banque de Sang Placentaire, CHRU de Nancy, F-54511 Vandoeuvre-Les-Nancy, France; 4 SSPC (Synthesis & Solid State Pharmaceutical Centre), V94 T9PX Limerick, Ireland; 5 Department of Chemistry, Royal College of Surgeons in Ireland, RCSI University of Medicine and Health Sciences, 123, St. Stephen’s Green, D02 YN77 Dublin, Ireland

**Keywords:** international mobility, Erasmus+, pharmacy curriculum, France

## Abstract

Internationalisation, as well as the need to interact with international partners in academia and in the pharmaceutical industry, brings an international experience to the pharmacist’s career, which is essential. The objective of present work is to provide a preliminary study of the current situation of the pre-professional mobility of pharmacy students. It represents the first case study of the international pre-professional mobility of pharmacy students in France, and in north-eastern France in particular. The study is based on a recent preliminary survey among pharmacy students, conducted in 2020 at the University of Lorraine’s Faculty of Pharmacy, reflecting the impact of international mobility programmes, such as the European Union educational and training mobility programme Erasmus+, on the pharmacy curriculum. The results of the present work tend to show that, despite a number of barriers to the international mobility of pharmacy students, the outcomes of international pre-professional mobility are rather positive in their globality.

## 1. Introduction

The pharmacist, as a health professional, plays an important role in public health. Nowadays, in the context of globalisation and facilitated international travel, accessibility to health services is a crucial issue. Any individual should have access to care and receive health advice and recommendations from a trained pharmacist who is qualified to manage a patient in an international context.

The upsurge of pandemics, including the ones of viral origin, with the topical example of COVID-19, shows the real issue of mobility. It highlighted the need to rely on mobile, multilingual health professionals with knowledge of the main healthcare systems, who work within international organisations such as the World Health Organisation (WHO), non-governmental organisations or humanitarian associations.

### 1.1. Historical Overview

Despite giving an image associated with a certain apparent sedentary lifestyle, the career of the pharmacist can be very itinerant. This mobility seems to correspond to current life and society, with specific requirements for each professional activity of the pharmacist [1,2]. There are many examples of professional mobility for a pharmacist. First, tenure-track pharmacists, who teach and conduct research, change universities, and laboratories during their careers. Then, pharmacists within the public service and public health authorities own a status that implies new assignments. Pharmacists in the pharmaceutical industry have a position that may lead to expatriation. Additionally, community pharmacists often move between cities or countries.

Indeed, this mobility has always existed, since the recognition of the pharmaceutical profession as an integral part of medical practice, starting from ancient times and passing through the Middle Ages, the Renaissance, the Crusades, the Cathars, and the Bogomils, it has spread throughout Europe, the Age of Discovery, colonialism, and the modern period [3,4,5,6,7,8,9,10,11,12,13]. In these eras, apothecaries frequently travelled because of wars or a taste for exploration, and for economic, personal, or professional reasons. In those periods, professional mobility was often based on two essential points of the profession: learning and settling as a pharmacist. This mobility of pharmacists seems to have perpetuated over the centuries and found its place in a globalised world and in a common European space that encourages mobility.

### 1.2. Pharmacists and Internationalisation

Nowadays, international mobility relies on strategy as it represents a growth engine for pharmaceutical companies and pharmacy-teaching academic structures in a global environment. The expansion of multinational companies, such as those in the pharmaceutical industry, can be seen as part of the internationalisation phenomenon. This trend favours the recruitment of candidates who have already experienced international mobility. Thus, young higher education graduates who have moved during their academic career in their pre-professional training often find themselves high on the list of companies during recruitment procedures, as observed by the Erasmus+ Higher Education Impact Study published by the European Commission in 2019 (European Commission, 2019) [14].

By doing so, internationalisation of post-secondary education fully impacts higher education and, consequently, the training of pharmacists [15,16]. One of the hallmarks of this internationalisation is the cross-border movement of students. In 2019, 5.1 million students were studying in an international mobility context, i.e., twice as many as in 2000 [17].

Student mobility is also stimulated and motivated by institutions, states, and supranational agencies such as the European Union (EU) with mobility programmes such as Erasmus+ [3]. Thus, international education, defined as the offer of education to international students, whether within national borders or abroad, is therefore seen as a value in itself [18,19].

### 1.3. Erasmus+ Programme

Erasmus+ is the programme created by the European Union to promote education and training in Europe. With a budget of EUR 14.2 billion for the period 2021–2027, Erasmus+ gives European citizens the opportunity to study, train, and gain experience abroad. This support allowed more than three million European citizens to spend part of their study years in a higher education institution or another developed organisation in another European country. Erasmus+ offers this possibility to students, trainees, workers, and others. Furthermore, Erasmus+ does not only involve Europe—it is a programme that expands worldwide.

The Erasmus+ Programme Guide 2021–2027 in the field of pharmacy education offers the same opportunities for studies and training abroad, as for any other curriculum. Nevertheless, the guidelines are applied differently by the pharmacy schools on intra-EU and even on a national scale, varying for one single year of mobility to several semesters throughout the entire pharmacy curriculum. For instance, periods abroad with Erasmus+ can last up to 24 months for study programmes that extend to one course only, such as architecture, medicine, and pharmacy.

### 1.4. The Benefits of the Pharmacist’s Mobility

Several international observations and studies have confirmed the advantages of pre-professional international mobility for students, including pharmacy students.

First of all, international mobility facilitates personal development. Mobile students are immersed in a different culture and way of life while living independently. Students quickly become more self-reliant, confident, and independent and develop a sense of initiative after their international experience [20,21,22].

International studies and internships also tend to facilitate professional development [20]. Among other healthcare students, pharmacy students are given the opportunity to observe and compare different healthcare practices between their host and home countries, with the possibility to learn different methods and approaches in the pharmaceutical field. This usually represents a unique opportunity to be inspired by innovative ways of practising pharmacy.

The exchange of good practices broadens and develops students’ academic knowledge, as well as their skills and qualifications. These elements improve their understanding and their knowledge of global pharmacy practices, allowing them to develop capacities that open up the possibility of working in the pharmaceutical field on a global level [20,21,23].

Another observation shows that studying internationally improves international mobility later in the career of future pharmacists [21,24]. Young graduates are able to make informed decisions about future work opportunities in the host country and internationally [24]. Social ties and international friendships created during international studies or internships can support their professional future, and students see their motivation increase when it comes to the possibility of considering future employment either in the host country or elsewhere [20].

Finally, studying abroad increases employability and improves career prospects [20,24]. The unique experience of these students allows them to become competent candidates for jobs in the various fields of pharmacy [25].

In addition to the benefits listed above, mobile students benefit from a number of additional advantages from their international experience. They experience the opportunity to meet new people, improve their language skills in English or the language of the host country, and boost their personal development. Indeed, these points reflect the twofold objective of the EU’s ambitious student mobility policy: enabling every EU citizen to communicate in two languages other than their mother tongue and promoting the participants’ identity as European citizens [26].

### 1.5. Challenges in the Pharmacist’s Mobility and the Brexit Case

The mobility of pharmacists as healthcare professionals has also raised a number of questions and debates lately, if only within Europe. The Brexit case is a very relevant example of a situation where professional labour mobility is significantly affected and student mobility reduced with the withdrawal of the UK from the Erasmus+ 2021–2027 framework.

EU directives force member states to accept the diplomas and qualifications of pharmacists from other member states [27]. Thus, European pharmacists can practise their profession anywhere in the EU, including in the UK. Even if there is not a licensure issue for practising pharmacy in the European Union (EU), partly to due to the Bologna declaration, there may be some barriers for pharmacists willing to practise outside of the EU [28,29]. For instance, the case of USA pharmacy practice, the pharmacists have to take an exam and receive a licence to practise. In these conditions, the pharmacy practice must obtain the Foreign Pharmacy Graduate Examination Committee (FPGEC) certification and a work visa. Some prior experience working as a pharmacist is usually necessary before practising abroad [30].

All of these changes have sparked several public debates about the important factors to consider when recruiting international pharmacists—language, professional practice, registration with the National Pharmacists Associations, and cultural integration. For instance, the remaining differences in the national pharmacopeia can be an issue and initiatives such as the European Pharmacopeia and the Pharmacopeial Discussion Group are engaged in the development of global quality standards and pharmacopeia harmonisation on international level. In the case of the United Kingdom, a number of studies have been carried out aiming at setting up measures to control the installation of European pharmacists in the country, and the regulations were strengthened and updated after the end of the Brexit transition period (31 December 2022) [31,32].

However, the tide can be reversed in certain situations for different reasons, which can sometimes be political, as in the case of Brexit. On the morning of 24 June 2016, thousands of European community pharmacists around the UK woke up to a drastically changed political landscape. This transition brought up numerous questions concerning the professional practice for non-British pharmacists and fore signs of restrictions, along with a feeling of uncertainty. This situation means that non-British pharmacists may be more willing to seek work at home and those still living in the UK consider returning in the near future [33]. This situation underlines the importance of international mobility in the career of a pharmacist and the need for flexibility in a moving global context.

### 1.6. Aim of the Study

The aim of this study was to evaluate the international mobility of the pharmacy students in the north-east part of France, where the geographical position should facilitate the international mobility given the closeness of the neighbouring countries (Germany, Belgium, Luxembourg, Switzerland). The impact of international mobility on the career projects of the students was also evaluated, in the context of a policy of internationalisation of third level education, stimulated by programmes such as Erasmus+, with its benefits and its challenges. The data collected intends to provide a preliminary snapshot of the current situation of the pre-professional mobility of the pharmacy students and provides a basis for a future in-depth study.

## 2. Materials and Methods

A recent preliminary survey (spring 2020) on the pre-professional mobility of pharmacy students conducted at the University of Lorraine’s (UL) Faculty of Pharmacy from 2013 to 2020 partly reflects the impact of Erasmus+ mobility on pharmacy students. With its 10,000 international students, the University of Lorraine is today the first French university in terms of number of outbound Erasmus mobility. The survey concerned the mobility profiles of 144 outbound/inbound students that had participated in exchange programmes (studies or traineeship). The data collection was conducted online and on the basis of centralised UL data. The online survey used the Google forms platform.

### 2.1. Study Design

This preliminary survey used a synthesised feedback from inbound and outbound Erasmus+ students from University of Lorraine on the studied topic. Participating students were invited to answer the anonymous questionnaire through a website link. Personal interviews were conducted to meet the need for further investigation.

Pre-survey was conducted to assess the validity of the survey by improving the clarity of your survey questions and of the proposed items.

The validity of the questions was assessed at two levels—once by two senior researchers and then tested by selected students. Final survey questions were modified based on the pre-survey analysis.

The questionnaire was composed of multiple-choice questions and single-choice questions. The questions in the survey took 10 to 20 min to complete.

In order to increase the response rate of the survey, the anonymity of the participants was ensured, the questionnaire was designed in a concise form, and the importance of the survey was highlighted.

The survey was managed using web panel through free online software (Google) and through invitations sent out by email. One email reminder was sent two weeks after the initial invitation.

### 2.2. Sample

A panel of 144 pharmacy students participating in the Erasmus+ programme (inbound/outbound, studies/traineeship) were used for this survey. These student profiles were selected due to their direct implication in the international student mobility programme. Demographics of the sample students, including gender, were also collected.

### 2.3. Data Collection

This preliminary survey was realised in three phases. The first phase included an initial international mobility University of Lorraine expert survey, which was followed by a survey of former Erasmus+ students’ experience abroad, and a survey of former Erasmus+ students’ career development.

The first phase was orientated towards international mobility experts, representatives from the University of Lorraine. That part of the study was designed to provide in-depth information on Erasmus+ student mobility on global UL level that could be compared to the results of the subsequent local pharmacy department surveys.

The second phase aimed to gather information on the personal value of an Erasmus+ mobility period abroad and to gather data about the mobility conditions on students’ personal level.

The third phase was directed to former Erasmus students already graduated and expected to be employed or in the labour market, with first professional experience. This phase was designed to gather information on the professional impact of the Erasmus+ mobility period.

For each survey, space was provided for pharmacists to provide justification for their answers, if needed.

### 2.4. Analysis

Based on participant responses, the questions were sorted according to mean scores and all the scores were summed and averaged by the total number of responses. Data were analysed through descriptive statistics.

All collected data were entered into a database and analysed mainly by using Excel (Microsoft Corporation. (2018). Microsoft Excel, Microsoft Corporation, One Microsoft Way, Redmond, WA 98052-7329, USA). Categorical data were expressed by percentage. Qualitative type data were thematically transcribed and analysed.

## 3. Results

### 3.1. Pre-Professional Mobility of European Pharmacists—The French Connection

**Figure 1:**
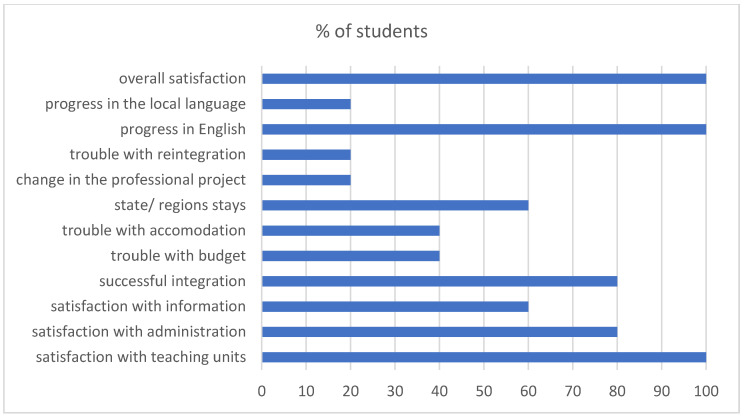
Feedback of students from Nancy involved in the Erasmus+ programme after their stay in 2018–2019.

Regardless of the level of academic success following international mobility, a clear difference is observed in the pharmacy students’ profiles upon their return. These students are much more open-minded; then, they quickly blend into the community life of the university and become very socially active (Figure 1).

The linguistic capacities of Erasmus+ students returning from their stay are clearly superior to those of their colleagues. Most of the students improve their English or their skills in the language of the host country—Spanish, Italian, and German in particular. This can be explained by the fact that the majority of pharmacy students select partner universities teaching in English for their pre-professional mobility. This observation reflects particularly well the effort of universities of Central and Eastern Europe to increase their attractiveness and strengthen their position in the EU academic world.

It is important to underline that all Erasmus+ pharmacy students are broadly satisfied by their mobility experience, including in the rare cases of academic difficulties (Figure 1).

Most of these students develop a taste for international mobility and wish to develop an international career. They also develop coping skills, along with a maturity level that sets them apart from their colleagues.

### 3.2. Career Path for UL Inbound Erasmus+ Pharmacy Students

**Figure 2:**
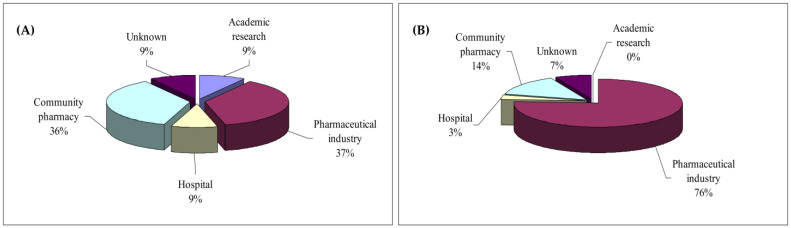
Comparison of first career pathways/jobs of (**A**) incoming international students, and (**B**) outbound students of the University of Lorraine’s Faculty of Pharmacy, who participated in an Erasmus+ mobility (studies or internship) between 2013 and 2016.

The beginning of the professional career of the young incoming Erasmus+ graduates in pharmacy is very varied, in terms of professional career paths. A majority find a placement in the pharmaceutical industry and community pharmacies, 37% and 36%, respectively. The hospital career and the academic career (research/teaching) are also present, at 9% each, and 9% of young graduates do not have a well-defined position (Figure 2A). The professional positions occupied are mainly of responsibility, such as Product Manager, Associate Area Manager, Lead Clinical Data Manager, Global Regulatory Affairs Specialist, Medical Science Liaison, and others. The employers are often large international pharmaceutical companies such as AstraZeneca, MSD, GSK Vaccines, Sandoz, and Takeda. It is important to underline the large proportion of young pharmacy graduates engaged in academic research, including in doctorate (Ph.D.) and clinical research, as well as in the community pharmacy career, as intermittent or full-time pharmacists.

**Figure 3:**
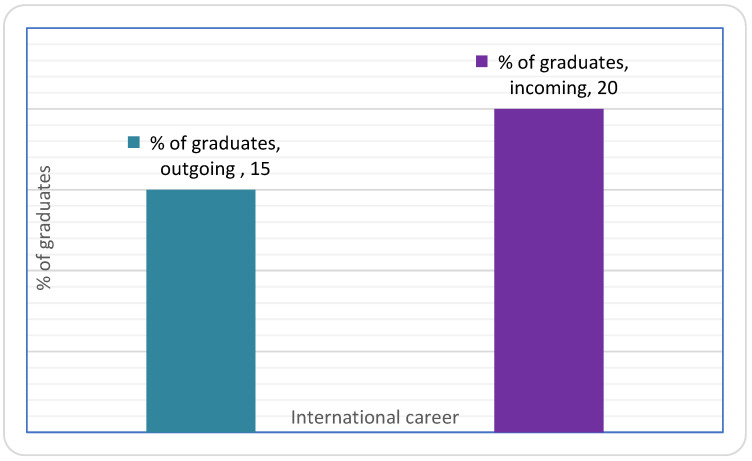
Proportion of outgoing and incoming young graduates (Erasmus+ mobility between 2013 and 2016) who found a job abroad.

Among the population of young graduates who made an Erasmus+ stay at the Faculty of Pharmacy of UL in the period 2013–2016, the majority found their first job in their country of origin (80%). The other 20% found an international job: 10% in France (Lorraine), 5% in another European country, and 5% outside Europe (Figure 3).

### 3.3. Career Path for UL Outbound Erasmus+ Pharmacy Students

**Figure 4:**
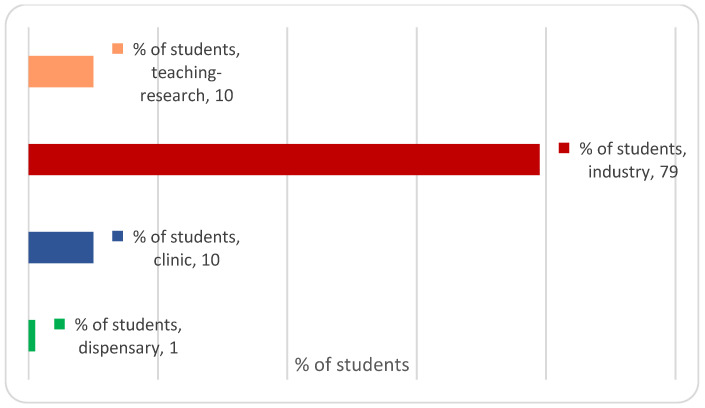
Specialisation choices of students in the University of Lorraine’s Faculty of Pharmacy who participated in an Erasmus+ mobility (studies or internship) between 2013 and 2016.

Upon returning from their Erasmus+ mobility, outbound pharmacy students from UL must make their choice of specialisation: academic research (teaching and research), community pharmacy (dispensary), pharmaceutical industry, or hospital pharmacy (clinic) (Figure 4).

A large majority of outgoing Erasmus+ students choose the pharmaceutical industry specialisation (79%), followed by the hospital and academic specialisations with 10%, and a minority will choose the community pharmacy specialisation (1%), as shown in Figure 4. Some of the outbound UL pharmacy students opt for a double course, pharmacy and engineering, as part of their curriculum. Thus, 27% of outbound UL Erasmus+ students will obtain a double degree in pharmacy and ENSIC (École Nationale Supérieure des Industries Chimiques, equivalent to National School of Chemical Industries) or ENSAIA (École Nationale Supérieure d’Agronomie et des Industries, equivalent to National School of Agronomy and Industries).

Very clearly, once graduated at the end of their 6th year of the UL pharmacy curriculum, the majority of young pharmacy graduates find the beginning of their professional career in the pharmaceutical industry (77%) with a significant diversity of positions held, from regulatory affairs and strategic marketing to quality control, production, and research and development (Figure 2B). The community pharmacy ranks second among jobs for young pharmacy graduates (14%), which is partly due to a number of graduates shifting their career path from the pharmaceutical industry towards pharmacy shortly after graduation. A minority occupies a hospital position (3%) and a certain number does not have a defined position (7%). It should be noted that academic research and teaching are not among the choices of the young UL Erasmus+ pharmacy graduates (Figure 2B). Among the population of young graduates who experienced an Erasmus+ stay in a partner university in the period 2013–2016, a large part (85%) found their first job in the country of origin (France): 15% in the region of Lorraine and 69% in another French region—mainly in the regions close to the cities of Paris and Lyon (Figure 3). The other 15% found an international job, in another European country (mainly Ireland, Switzerland, Sweden, and Serbia). The job profile occupied by the young UL pharmacy graduates often corresponds to a position of responsibility. Positions include posts such as Director Regulatory Programme, Business analyst, Technical Manager, Regulatory Affairs Manager, Product Manager, Quality Control Manager, International Clinical Studies Coordinator, and Clinical Research Associate. Among the employers of young graduates are large international companies, start ups, and public bodies, such as Sanofi, Roche, Pfizer, Mylan, Lonza, GSK, Hospices Civils à Lyon, and others.

## 4. Discussion and Perspectives in Pharmaceutical Mobility

In summary, this survey shows the importance pre-professional mobility has on pharmacy students. Notwithstanding the barriers that the pharmacy students have to face during the preparation of the mobility project and the difficulties that have to be overcome during the mobility, the administrative, logistic and other issues, the large majority of the mobility students are satisfied with their experience abroad, including the academic achievements and linguistic progress. An important part of these students’ choices to work and develop their professional project abroad at the end of their academic curriculum, with few academic careers; a large portion of community pharmacy, and mostly industrial pharmacy careers, lead to managerial and executive positions.

In this exact context, the European Erasmus+ mobility programme proves to be an essential tool to bring an international dimension to future pharmacists’ careers. Indeed, according to the impact study of the Erasmus+ programme carried out by the EC in 2019 (European Commission, 2019), students who complete an Erasmus+ mobility to study train and improve their skills in terms of employability [14,34]. A large majority (72%) say it was beneficial or very beneficial for them to find their first job. Their mobility increased their technical, interpersonal, and intercultural skills and qualifications, as well as their self confidence, their ability to achieve their goals, and their social and cultural openness. Around 40% of participants who have completed an internship have been offered a job in the companies/organisations in which they have completed their internship, around 10% have started their own business, and many more (75%) plan to do so in the future. Erasmus+ participants have a clearer idea of what they want to achieve in their future careers after their stay abroad. Those in the labour market report being happier with their work than students who did not join the Erasmus+ programme. Their job is also more likely to be located abroad (23% of Erasmus+ students, compared to 15% for the other students) or to have an international dimension.

Students who choose international mobility during their pharmacy studies often gain experience of high added value both on a personal level and concerning their future careers as healthcare professionals. In the context of increasingly rapid globalisation, future pharmacists must bring international expertise in drugs and pharmacology and must be able to communicate with their peers and the patients in a variable linguistic context, considering differences not only at the level of the health system but also cultural ones.

Good co-operation between healthcare professionals at the international level is the basis for better patient care on all fronts—from basic academic pharmaceutical research to industrial development and clinical application, up to drug dispensing and patient counselling in the community pharmacy.

According to most human resources specialists, this added value, which comes with a multicultural experience, is seen as a major asset that simplifies job searches. A candidate who presents transferable and transversal skills they acquired during an internship or studies abroad is perceived by recruiters as an active, determined, and independent person. This kind of candidate is often ready to take on challenges and show flexibility and willingness in the face of new situations under demanding circumstances. An experience abroad is also a way to acquire international adaptability, intercultural sensitivity, and self confidence by effectively learning to share ideas—qualities seen as an advantage for a future professional career.

The European trend which shows that Erasmus+ students are more successful at finding an international job is also confirmed in the pharmaceutical field. The analysis proves that Erasmus+ students are more likely to find a job rapidly after graduation. This observation also applies to pharmacy students following pre-professional mobility, after which the majority of young graduates occupy positions of responsibility in their field of expertise in their home country or abroad.

The new Erasmus+ programme of the European Commission and the recommendations of the European Union set a target of 20% of higher education graduates benefiting from transnational mobility. Regarding the international mobility of pharmacy students, whether for studies or internships, students seem on the whole interested, but the final rate of mobility does not yet reach the threshold set by the European Union.

Indeed, nowadays pharmacy students face several difficulties concerning the possibility of doing part of their studies abroad and often struggle when trying to access international exchange programmes, such as Erasmus+. Some of the challenges lie in the fact that student mobility for regulated professions such as pharmacy can be more difficult and the pharmacy schools are not really stimulating, and sometimes discouraging, students to study abroad, accentuating the lack of fulfilment of the curriculum criteria and on the recognition of the studies abroad. Based on local observations, it seems that there are several hurdles to overcome as far as academic structures are concerned. It is necessary to make the pharmacy training system more flexible with regard to international exchanges, by overcoming prejudices and stereotypes regarding Erasmus+ mobility, which are relatively common phenomena, especially in smaller structures. This argument works in favour of all the efforts to also improve mobility for administrative and academic staff so that these persons can also properly appreciate the challenges and advantages of international professional mobility, as they have not had the opportunity to do so until now [35,36]. In the academic context, international mobility is also considered as an unfavourable practice to the phenomenon of “academic inbreeding”, which persists as a local challenge and a global problem, especially for smaller structures and universities [37,38].

In terms of pharmacy training, any restriction of pre-professional mobility for studies to one single year in the curriculum does not promote the full deployment of the mobility programme. It should be underlined that a larger part of pharmacy students should be allowed to access international pre-professional mobility. In that sense, it seems important that all levels and streams of the pharmacy curriculum—community pharmacy, industry, and clinic/hospital orientations, including the years of the pharmacy thesis– remain open to pre-professional mobility. Students should be encouraged to opt for study and internship mobility during their pharmaceutical training, not only by their university but also by the faculty.

Among various measures that are currently tested at the Faculty of Pharmacy of the University of Lorraine to encourage and facilitate international mobility, it is important to highlight the creation of student unions and associated structures. Their goals include enabling linguistic learning, supporting inbound international students, facilitating communication, and organising workshops that prepare students for international mobility, in addition to regular monitoring procedures for outbound students. Cultural exchange workshops with international students and workshops for sharing positive experiences following international mobility also contribute to strengthening student mobility. These measures include the expansion and development of the network of international partners, aimed at providing a very wide range of destinations, specialisations, and languages of learning to pharmacy students. This expansion has permitted a very wide geographical coverage of destinations in Europe and neighbouring countries, with an opening to partners of high academic level in terms of teaching and research, who usually offer multilingual training, including in English.

It is also essential to increase international exchanges for studies and internships with partner countries beyond the borders of the EU, such as Russia, Georgia, the United States, Canada, as well as countries of Latin America with traditions in pharmacy, innovative pharmacy education, and a rapidly developing pharmaceutical industry. Indeed, programmes such as International Credit Mobility and Blended Intensive Programmes allow the mobility under the Erasmus+ framework, while programmes such as BCI (*Bureau de Cooperation Interuniversitaire*) and SEP (Student Exchange Programme) give the opportunity for exchanges with non-EU partner institutions. Interaction with these new partners and the founding of joint international pre-professional training projects would better prepare pharmacy students as future health professionals in the even more dynamic world of tomorrow.

The missions of pharmacists are currently evolving in order to ensure better patient care. This progress is specific to each and every country and thanks to international mobility, pharmacists can be trained using innovative methodologies and approaches. Through their expertise in medicines on a global level and their linguistic capacities, they subsequently contribute to optimal care of patients, both in clinical and ambulatory conditions.

There are, undoubtedly several limitations and caveats that must be considered for the data collection and the analysis of the dataset. The survey was limited by the fact that some of the participants were hard to reach, with already inactive UL mailing addresses, and its reliability may be methodologically questioned due to the limited number of participants and the short time lapse of the survey (spring 2020). Second, the quality of the survey was dependent on the quality of online recruitment of the participants. Third, using online mode only excludes part of the student population by design. These issues imply that the web-experienced student population might be over-represented in this survey.

## 5. Conclusions

The present work is the first case study of the international pre-professional mobility of pharmacy students in France, and in the north-eastern France in particular. The low number of pharmacy students opting for international mobility during their academic curriculum is representative of the lack of stimulation from the academic structures/schools of pharmacy and it is clear that in the case of pharmacy students the internalisation of third level education is not as easy, compared to other study programmes. Indeed, there are a number of barriers to the international mobility for the pharmacy students, such as the differences in the pharmacy curricula among the partner schools, the difficulties with the information collection/accommodation in the host university, and the sometimes negative attitude towards the international exchanges from the home institution, etc., leading to the general feeling in the majority of the pharmacy students that it is hard to participate in international mobility projects. However, the results of the present work tend to show that the outcomes of international pre-professional mobility are rather positive in their globality for the few pharmacy students that participate in the exchange programmes.

Requiring international mobility within the framework of pre-professional pharmacy mobility could effectively promote a first experience outside the national context for many students. In that sense, it is important that universities encourage and facilitate student international mobility starting from the first course of study. International placements and mobility throughout the pharmacy curriculum should represent an important goal for all the European pharmacy faculties in order to ensure higher educational standards. The advantages of international mobility programmes can contribute to developing competent and confident pharmaceutical healthcare professionals in a globalising world.

## Data Availability

Not applicable.
